# Supplementary material for: New flow relaxation mechanism explains scour fields at the end of submarine channels
Source: Nat Commun. 2019 Sep 27;10:4425. doi: 10.1038/s41467-019-12389-x (PMC6764982; doi:10.1038/s41467-019-12389-x)
Supplement: Supplementary file 1 — Supplementary Information [file 41467_2019_12389_MOESM1_ESM.pdf]

Supplementary Information for

New flow relaxation mechanism explains scour fields at the end of submarine channels

by

Pohl et al.

Supplementary Figures

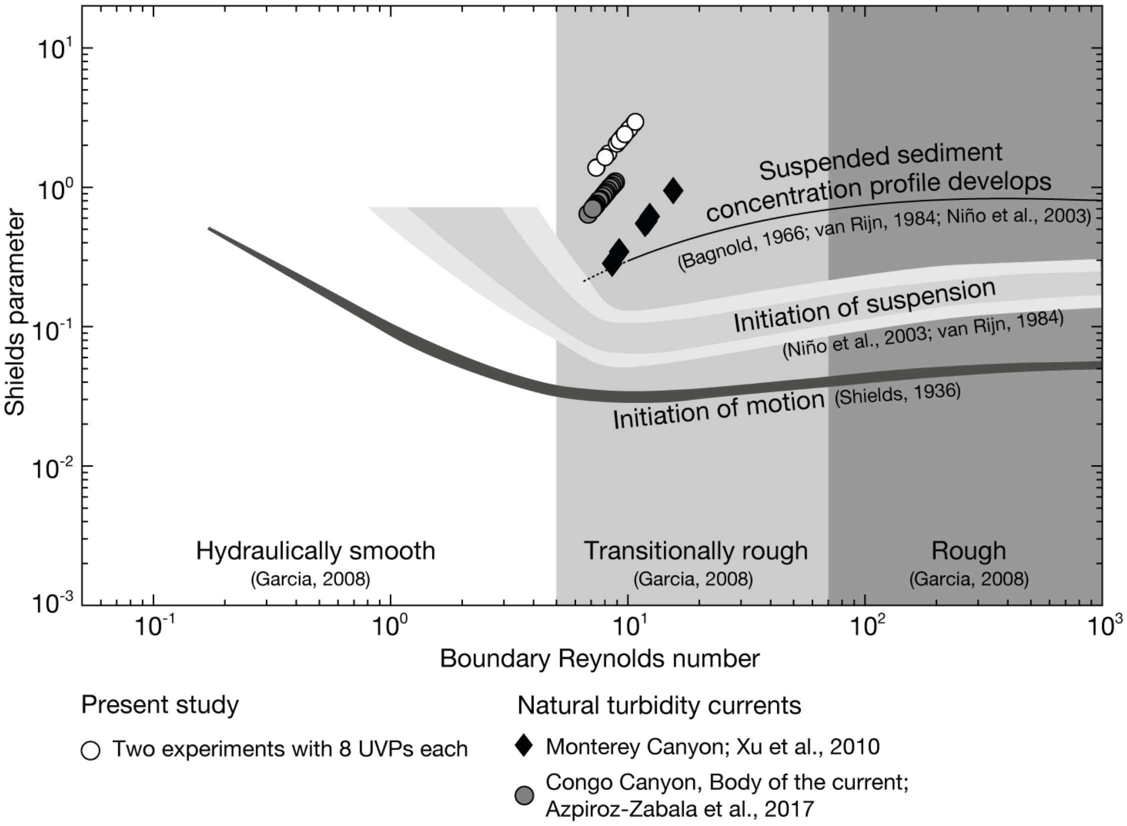

Supplementary Figure 1. Shields mobility diagram. Describes the dominant sediment transport mode for a given set of hydrodynamic conditions. Modified after [1] and [2]. Natural flows were monitored in the Monterey Canyon [3], and the Congo Canyon [4]. For calculation of the Congo Canyon, the body of the current and a grain size of 200 µm was used. Regime boundaries after: [1,5–8]

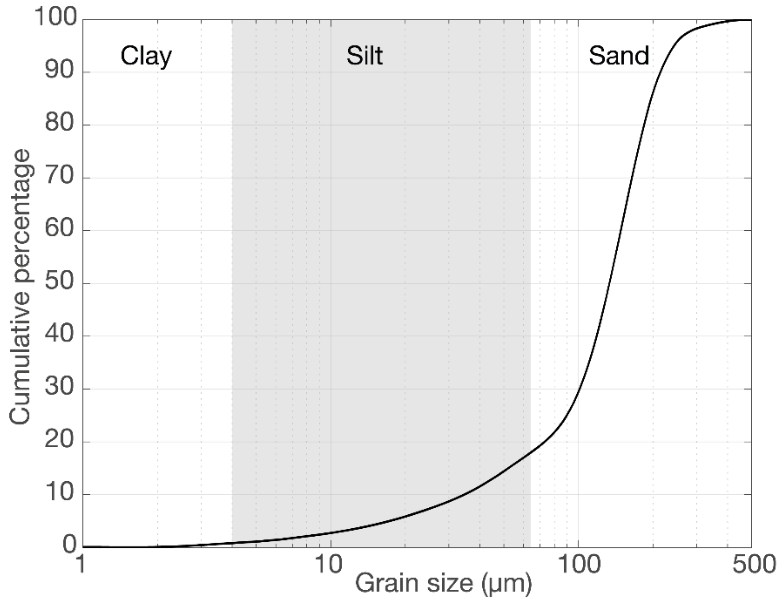

Supplementary Figure 2. Cumulative grain-size distribution. Sand of identical grain size was used for the floor of the flume tank and for the suspended sediment of the turbidity current. Grain size was measured with a laser particle sizer (Malvern Mastersizer 2000).

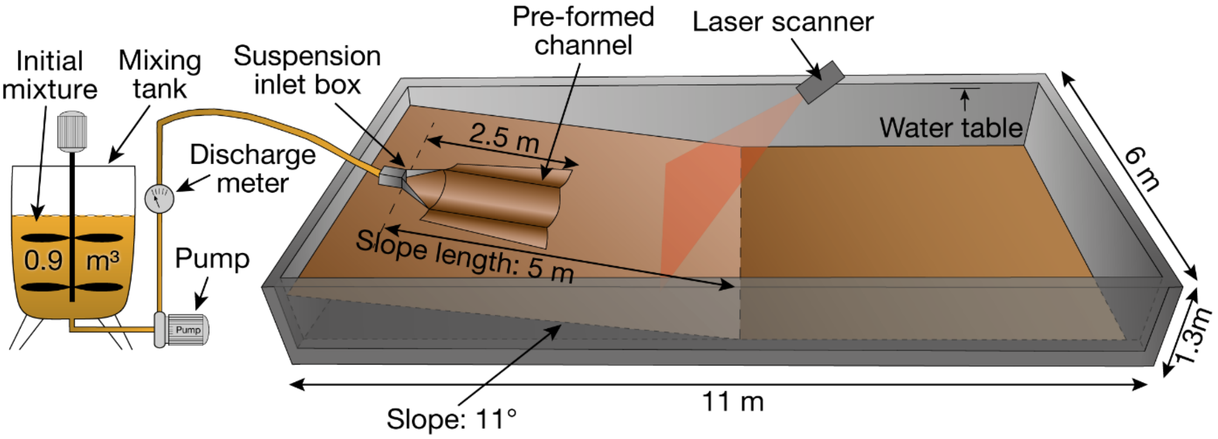

Supplementary Figure 3. Schematic drawing of the experiment setup. Note that the length of the reference (no loss of confinement) experiment extended 5 m further downslope.

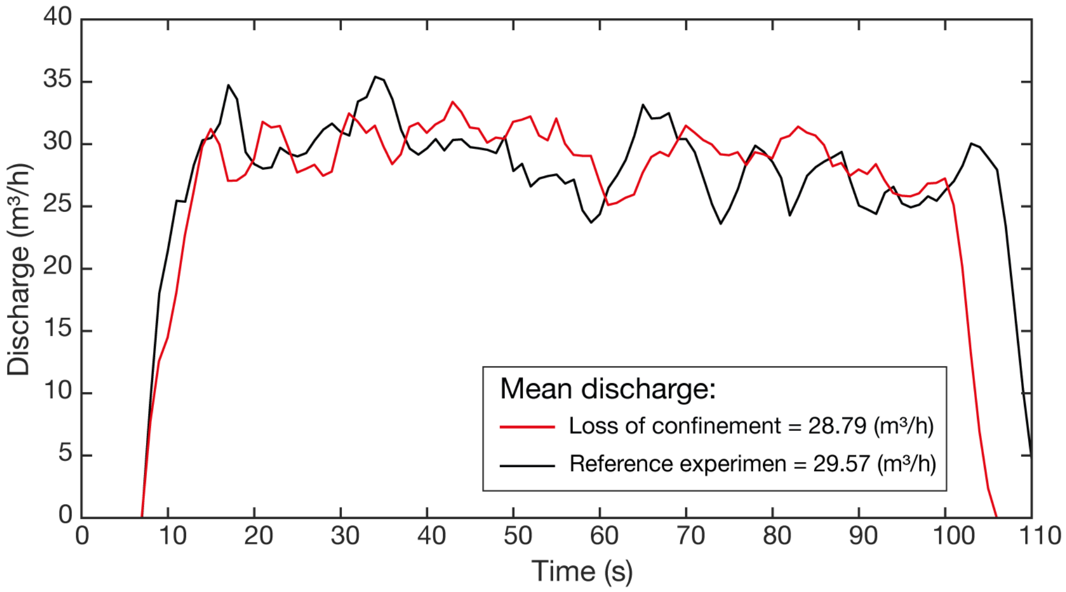

Supplementary Figure 4. Discharge measurements of the two experiments. The discharge was measured with an electromagnetic flow-meter (Krohne Optiflux 2300). The mean discharge was calculated by averaging over the time interval between 15 to 95 s.

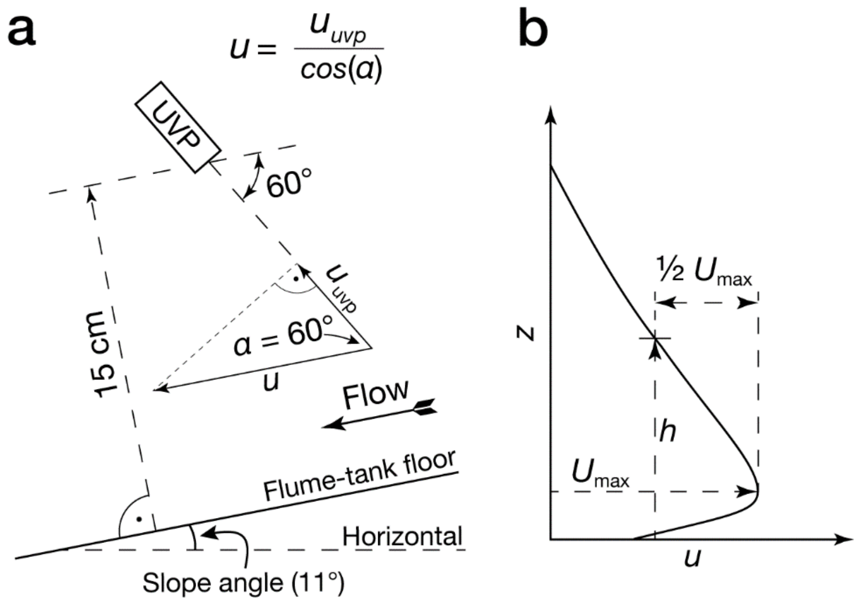

Supplementary Figure 5. UVP orientation and parameterization of the velocity profile. a,**b**, (**a**) The orientation of the UVP and the trigonometric calculation to calculate bed-parallel velocities. u_{UVP} is the velocity component directed toward the UVP and u is the bed parallel velocity in downflow direction. Not to scale. (**b**) Sketch of a velocity profile illustrating the analysis of the time-averaged velocity profiles. Redrawn from [9].

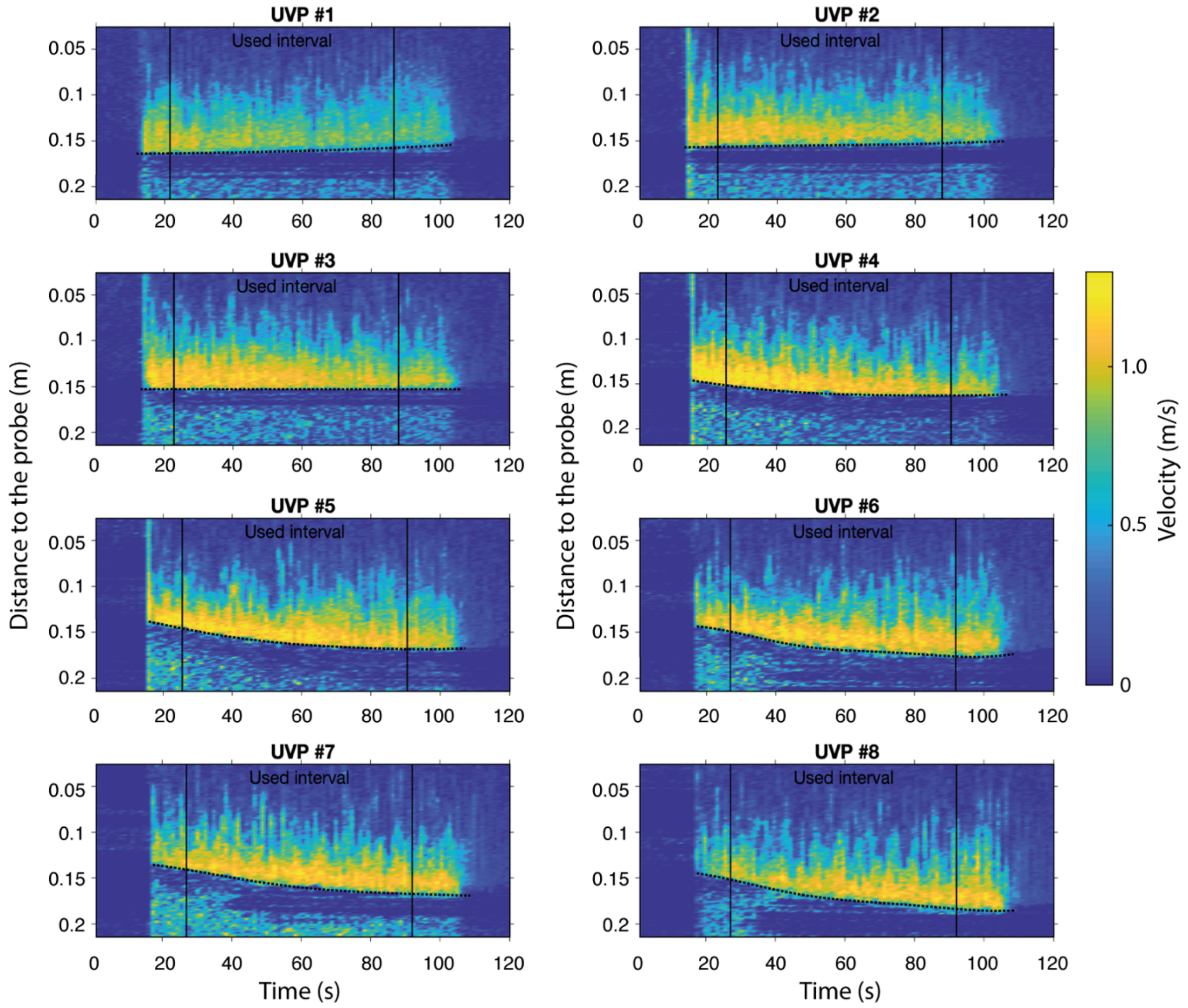

Supplementary Figure 6. Velocity measurements in the experiment with loss of confinement. The solid vertical lines mark the interval that was used for analysis of the velocity data. The dashed line indicated the position of the bed, where a sharp decrease in velocity occurs.

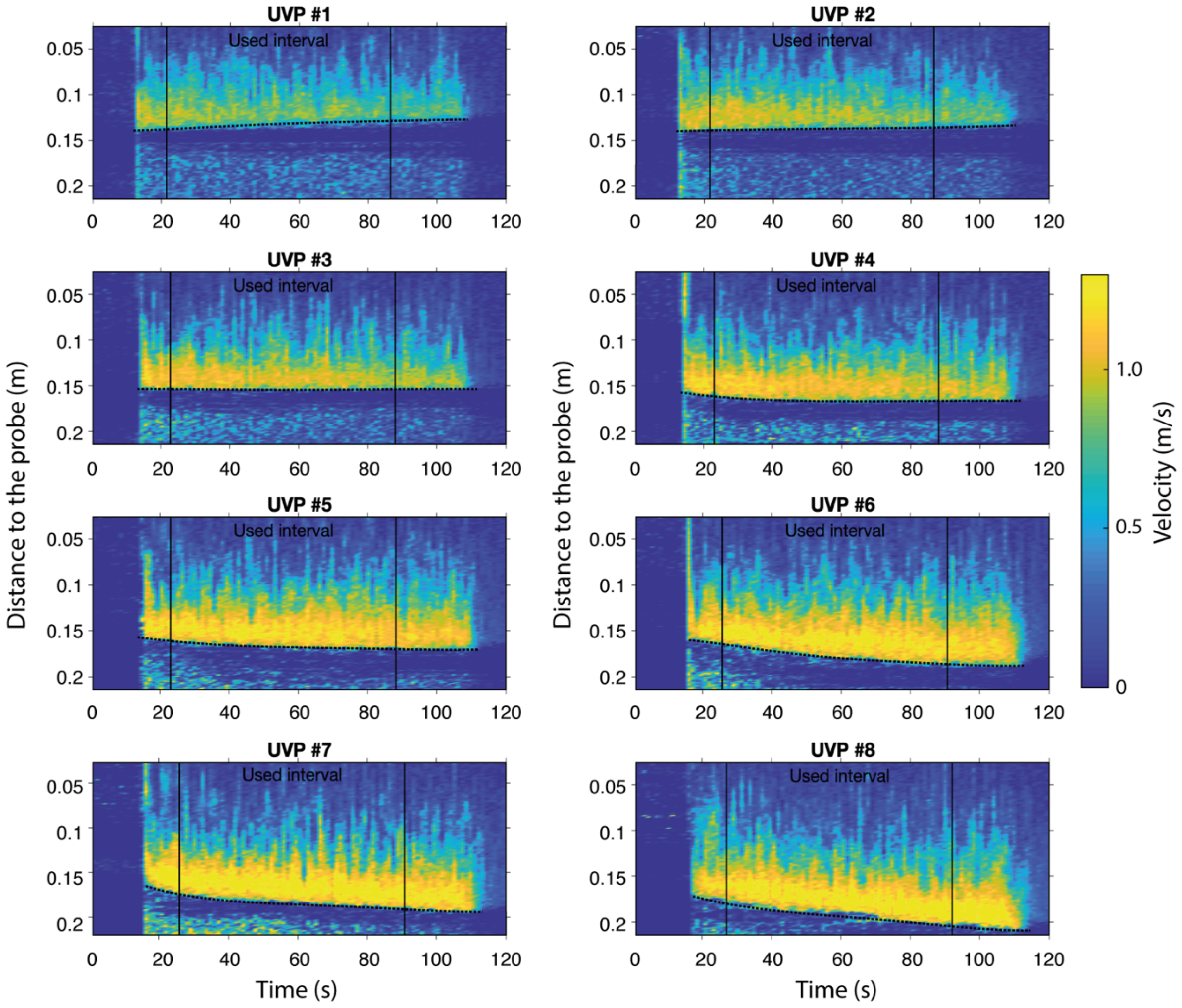

Supplementary Figure 7. Velocity measurements in the reference experiment. The solid vertical lines mark the interval that was used for analysis of the velocity data. The dashed line indicated the position of the bed, where a sharp decrease in velocity occurs.

Supplementary Tables

UVP acquisition settings

Manufacturer and type	MET-FLOW; DUO MX
Speed of sound in water (m/s)	1480
Measurement window (mm)	246.79
Number of channels	235
Distance between channel centers (mm)	0.925
Channel width (mm)	3.7
Frequency of the ultrasound beam (MHz)	1
Number of cycles per pulse	5
Number of sound pulses per measurement	32
Minimum on-axis velocity (mm/s)	-1081.9
Maximum on-axis velocity (mm/s)	1073.4
Velocity resolution (mm/s)	8.5
Time between each measurements (s)	1.247

Supplementary Table 1.

Supplementary References

1. Shields, A. Anwendung der Aehnlichkeitsmechanig und der Turbulenzforschung auf die Geschiebebewegung. *Mitteilungen der Preußischen Versuchsanstalt für Wasserbau und Schiffbau* **26**, (Technische Hochschule Berlin, 25 pp, 1936).
2. de Leeuw, J., Eggenhuisen, J. T. & Cartigny, M. J. B. Morphodynamics of submarine channel inception revealed by new experimental approach. *Nat. Commun.* **7**, 1–7 (2016).
3. Xu, J. P. Normalized velocity profiles of field-measured turbidity currents. *Geology* **38**, 563–566 (2010).
4. Azpiroz-Zabala, M. *et al.* Newly recognized turbidity current structure can explain prolonged flushing of submarine canyons. *Sci. Adv.* **3**, 1–12 (2017).
5. Bagnold, R. A. *An Approach to the Sediment Transport Problem from General Physics. USGS Professional Paper 422-1* (USGS Professional Paper 422-1, U.S. Government Printing Office, 1966). doi:10.1017/S0016756800049074
6. Nino, Y., Lopez, F. & Garcia, M. Threshold for particle entrainment into suspension. *Sedimentology* **50**, 247–263 (2003).
7. van Rijn, L. C. *Principles of sediment transport in rivers, estuaries and coastal seas*. *Aqua publications* (Aqua publications. 790 pp., 1993). doi:10.1002/9781444308785
8. Garcia, M. Sedimentation Engineering: Processes, Measurements, Modeling and Practise. *Am. Soc. Civ. Eng.* 1132 pp. (2008).
9. Launder, B. E. & Rodi, W. The Turbulent Wall Jet Measurements and Modeling. *Annu. Rev. Fluid Mech.* **15**, 429–459 (1983).
